# Observations on the Dysentery of the West Indies, with a New and Successful Manner of Treating It

**Published:** 1781-08

**Authors:** 


II. *Obfervations on the Dyfentery of the Weft Indies, with a new and fuccefsful manner of treating it. By* Benjamin Mofeley, *furgeon at Kingfton in Jamaica.* 4to. Kingfton, 1780. 20 pages.

THE dyfentery is a difeafe fo deftructive to foldiers in camps and garrifons, and fo conftant an attendant on all military operations

The great Sydenham, to whom the world is indebted for an inestimable work founded in nature, on a basis applicable to every climate, discovered the dysentery to be a fever of the season, or *sui generis* 'turned inwards on the 'intestines.' In the course of twelve years experience in Jamaica, and from every account he has been able to procure from different parts of the West Indies, Mr. Moseley has invariably had occasion to be convinced of the truth of Sydenham's opinion, and has remarked, that "as the flux distinguishes by the number of

" ftools the quantity, fo it does the ftate of the " fever of the feafon, when it prevails, or of " the fubject difeafed; the ftools being moft " frequent at thofe hours when fevers are in " their exacerbation." He is perfuaded that this fever, like moft others, is caufed by obftructed perfpiration; not confined to cold, hot, wet, or dry feafons; particular food, water, liquors, or fruit; but chiefly depending on fudden tranfitions, and fuch other caufes, as expofe people to have this difcharge haftily ftopped. Having ever adapted his practice to this principle, fo he affures us he has feen the complaint conftantly relieved, by bringing back that difcharge to its natural channel, and that he has feldom found it difficult to remove it fpeedily, when taken in the beginning. He very properly condemns the practice of attacking the diforder in the bowels with opiates and aftringents, and obferves, that Dovar's powder and other diaphoretic medicines recommended for the flux have hitherto been exhibited in fuch a manner as often to have produced more harm than good; a clear proof, he thinks, that the fkin has not been really looked to for relief. He afcribes the good effects of ipecacoanha and antimonials in this difeafe folely to their increafing the determination to the fkin.

Speaking of emetic tartar, he remarks, that it is in many reſpects a dangerous medicine (except merely as an emetic) in hot climates, where the nervous ſyſtem is ſo extremely irritable, and that when "injudiciouſly given to young, irritable, plethoric people, in the beginning of a fever, and previous to proper evacuations, inſtead of exciting a diaphoreſis, a ſpaſm is produced in the ſtomach; inceſſant vomiting; inflammation; the veſſels of the thorax and head are ſtifled with blood, and the patient vomits away his life." In a note to this paſſage the author obſerves, that experience has ſhewn the ill conſequence of vomits in the inflammatory fever which Europeans are ſubject to on their arrival in the Weſt Indies; and that it is eaſily cured by bleeding and cathartics, frequently repeated; and in general by a cooling regimen.

After offering ſome remarks on the ſituation and conſtruction of hoſpitals, and condemning the little huts uſed for this purpoſe in Jamaica, where the patient is placed at a door-way, or raiſed on a platform to the level of an open window to prevent ſuffocation, our author proceeds to illuſtrate his ſubject by a ſhort account of the dyſentery, as it appeared at the time of his writing, (May 1780), amongſt the troops in that iſland, together with his method of treating it. The diſeaſe, we are told, was not prevalent in the camp at Fort Caſtile, the ſituation of which was in a riſing, airy ſituation near the ſea, free from ſtagnant water and unwholeſome exhalations, but expoſed to the force of all the elementary tranſitions.

The general ſymptoms were a chillneſs in the beginning, ſucceeded by feveriſh heats; gripings, and frequent ſmall motions; ſickneſs of the ſtomach, and ſometimes retchings; theſe were ſoon followed by copious purging, with green, brown, or yellow watery ſtools, mixed with or ſucceeded by great diſcharges of blood. The ſtools varied in fœtor and appearance, according to the periods of the diſeaſe, or as they were more or leſs retained. In ſome the diſeaſe was preceded and accompanied by a conſiderable degree of fever; in others the pulſe was but little affected. In the laſt ſtages of the diſeaſe the patient was continually harraſſed, particularly at night, with ſmall, bloody, ſlimy ſtools; the tongue was greatly furred, and ſometimes of a brown or black colour; apthæ appeared but ſeldom.—This was the general account of thoſe who experienced the violence of the diſeaſe, and

ſurvived

ſurvived the firſt week; but many who were ſeized at the beginning of the epidemic periſhed within that time. The curative indications, according to our author, were to cauſe a revulſion to the ſurface of the body, and to cleanſe the inteſtines. He generally began with bleeding the patient, after which he preſcribed a vomit of ipecacoanha and an opiate. The bowels were then evacuated by a ſuitable doſe of James's powder, which, at the ſame time that it effectually cleanſed the primæ viæ, never failed, we are told, to excite a plentiful ſweat.

The revulſion having been thus begun, was completed by ſudorifics. This was effected by uniting the Thebaic tincture with antimonial wine, and adminiſtering them as occaſion required. The author remarks, that it is always neceſſary in the flux, when a ſweat is intended by antimonial or other emetic medicines in ſmall doſes, to add opium to take off their irritation; by which means their doſes and effects may be greatly extended.

When the diaphoreſis was begun, the patient was covered with a blanket, and care taken that the cool air ſhould not be admitted directly upon him; at the ſame time he was plentifully ſupplied with warm diluting liquors.

 Mr.

Mr. Moſeley obſerves, that when the ſudorific proceſs has been ſucceſsfully continued, all the ſymptoms grow milder; and if an eruption appears on the ſkin the diſeaſe ſoon vaniſhes. But, on the contrary, if the flux continues obſtinate and the ſweats go on kindly, it will not only be requiſite to carry off the morbific humours by a doſe of the antimonial purgative, but repeated vomits of ipecacoanha are to be given.

Another cauſe of obſtinacy in the flux are indurated fœces lodged in the inteſtines, for which reaſon, when the ſweats have been plentiful, the pulſe moderate, and the flux ſtill continues troubleſome, we are adviſed to repeat the antimonial purgative.

Our author purſues this method, repeating it as occaſion may require, till the patient is in a condition to take the bark, and other tonics.—When the flux, as will ſometimes happen, continues troubleſome from mere weakneſs, without any material gripings or feveriſh ſymptoms, he never heſitates to give bark with ſnake-root and wine. He cautions us, however, againſt the uſe of bark in ſubſtance in theſe caſes, as it cauſes irritation and griping, and endangers a return of the diſeaſe. A ſtrong decoction of it therefore is to be preferred.

As

As the flux is always increafed at the approach of night, fo for fome time after it has abated the patient grows feverifh in the evening. On this account we are advifed to defift from the bark, and give a gentle diaphoretic at night. The remaining acrimony, which fometimes keeps up a flight irritation after every other fymptom is removed, may be corrected by abforbents, and carried off before the ufe of the bark; or at any fubfequent period, if it fhould recur, with rhubarb and magnefia, or any mild cathartic. During the convalefcent ftate of thofe who have been much reduced, and to prevent a relapfe, a flannel jacket is recommended to be worn next the fkin.

Mr. Mofeley remarks, that in fome cafes, when the attack is fudden and violent, it will be neceffary to give opiates and cordials, in order to "arreft the hurry of the difeafe; to procure "time to put fome rational means of cure into "execution; to give other medicines their in-"tended effect, and to eafe thofe *tormina*, which "are otherwife intolerable." In any other fituation, however, he condemns the ufe of opium, given alone and continued for any length of time, as being likely to produce the moft baneful effects.

In an advertisement prefixed to the work, the author informs us, that as the practice of military hospitals in Europe is not suitable to the seasons and climate of the West Indies, he intends shortly to communicate some observations he is furnished with, on the cure and treatment of other diseases incident to the army.

